# A Bacterial Artificial Chromosome Reporter System for Expression of the Human *FOXP3* Gene in Mouse Regulatory T-Cells

**DOI:** 10.3389/fimmu.2017.00279

**Published:** 2017-03-13

**Authors:** Masato Tsuda, Yukiko Tone, Chihiro Ogawa, Yoshiko Nagaoka, Makoto Katsumata, Andra Necula, Duncan Howie, Esteban Masuda, Herman Waldmann, Masahide Tone

**Affiliations:** ^1^Department of Biomedical Sciences, Cedars-Sinai Medical Center, Los Angeles, CA, USA; ^2^Sir William Dunn School of Pathology, University of Oxford, Oxford, UK; ^3^Rigel Pharmaceuticals, South San Francisco, CA, USA

**Keywords:** FOXP3, regulatory T cells, gene expression, transcription factor, bacterial artificial chromosome

## Abstract

The transcription factor FOXP3 plays key roles in the development and function of regulatory T cells (Treg) capable of preventing and correcting immunopathology. There has been much interest in exploiting Treg as adoptive cell therapy in man, but issues of lack of nominal antigen-specificity and stability of FoxP3 expression in the face of pro-inflammatory cytokines have been a concern. In order to enable fundamental studies of human *FOXP3* (*hFOXP3*) gene regulation and to provide preclinical tools to guide the selection of drugs that might modulate hFOXP3 expression for therapeutic purposes, we generated hFOXP3/AmCyan bacterial artificial chromosome (BAC) transgenic mice and transfectants, wherein hFOXP3 expression was read out as AmCyan expression. Using the transgenic mice, one can now investigate *hFOXP3* gene expression under defined experimental conditions used for mouse Foxp3 (mFoxp3) studies. Here, we demonstrate that *hFOXP3* gene expression in BAC transgenic mice is solely restricted to CD4^+^ T-cells, as for *mFoxp3* gene expression, showing that hFOXP3 expression in Treg cells depends on fundamentally similar processes to mFoxp3 expression in these cells. Similarly, hFOXP3 expression could be observed in mouse T-cells through TCR stimulation in the presence of TGF-β. These data suggest that, at least in part, cell type-specific human and mouse *foxp3* gene expression is regulated by common regulatory regions which for the human, are located within the 110-kb human FOXP3 BAC DNA. To investigate *hFOXP3* gene expression further and to screen potential therapeutics in modulating *hFOXP3* gene expression *in vitro*, we also generated hFOXP3/AmCyan expression reporter cell lines. Using the reporter cells and transcription factor inhibitors, we showed that, just as for mFoxp3 expression, inhibitors of NF-κB, AP1, STAT5, Smad3, and NFAT also block hFOXP3 expression. hFOXP3 induction in the reporter cells was also TGF-β dependent, and substantially enhanced by an mTOR inhibitor, Torin1. In both the reporter transgenic mice and cell lines, histone H4 molecules in the hFOXP3 promoter and enhancers located in human CNS1 and CNS2 regions were highly acetylated in natural Treg and TCR/TGF-β-induced Treg, indicating *hFOXP3* gene expression is regulated by mechanisms similar to those previously identified for the *mFoxp3* gene.

## Introduction

Regulatory T cells (Treg) are essential for preventing autoimmune disease and other forms of immunopathology (1–3). Their development and function is regulated by a transcription factor Foxp3 (1, 4). Due to the availability of genetically modified/disease-model mice, major efforts investigating *foxp3* gene expression have been conducted in mice. However, recent studies have suggested that mouse and human *foxp3* genes might be subject to different regulatory mechanisms (5–14), suggesting that mouse data may not necessarily guide clinical relevance. There has been much interest in exploiting natural or thymic-derived regulatory T-cells (nTreg or tTreg) as adoptive cell therapy in man, but issues of antigen-specificity and stability of FoxP3 expression in the face of pro-inflammatory cytokines have been a concern (15, 16). As antigen-specific CD4^+^ Foxp3^+^ Treg can be induced *in vitro* (iTreg), it has also been considered desirable to acquire expanded, stable antigen-specific populations of Treg, as efficient mediators of suppression (15, 17). So as to enable further fundamental studies of *hFOXP3* gene regulation, and to provide preclinical tools to guide the selection of drugs that might modulate hFOXP3 expression for therapeutic purposes, we generated hFOXP3/AmCyan bacterial artificial chromosome (BAC) transgenic mice as well as transfectants into a murine T-cell line, wherein hFOXP3 expression could be investigated through AmCyan expression.

Mouse *Foxp3* (*mFoxp3*) gene expression is regulated by TCR signaling, IL-2, and TGF-β through at least four distinct regions, a promoter and elements located in three CNSs (conserved non-coding sequence), CNS1, CNS2, and CNS3 (18–30). The *mFoxp3* promoter is located upstream of the non-coding exon, and its activity is regulated by several transcription factors activated through TCR signaling, including AP1 (22), cRel (26), and FOXO1/3 (18). This promoter activity is relatively weak, and cell type-specific expression of *mFoxp3* gene is cooperatively maintained by enhancers located in CNS1 and CNS2. TGF-β and IL-2 signaling response elements are located in the CNS1 and CNS2 regions, respectively (28, 31–33). We have identified an enhancer (Enhancer1) in the CNS1 region, and Enhancer1 is activated by signaling through the TGF-β receptor and TCR *via* Smad3 and NFAT, respectively (28). Subsequently, Xu et al. (29) have shown that AP1 and retinoic acid also regulate the activity of this enhancer. Another enhancer (Enhancer 2) is located in CNS2 and functions as an IL-2/TCR response regulatory region (19, 31). Foxp3 expression and Treg function are also regulated by DNA methylation (34, 35). Indeed, this CNS2 enhancer is negatively regulated by DNA methylation through a CpG island that is highly methylated in Foxp3^−^ cells, but demethylated in Foxp3^+^ Treg. Several transcription factors activated by TCR signaling bind to this enhancer (19, 31, 36), yet as activities of these factors are DNA methylation sensitive, it is unclear how the highly methylated enhancer becomes activated. Recently, we proposed that activated STAT5 generated through TCR/TGF-β/IL-2 signaling pathways binds to the methylated CNS2, so enabling enhancer activity (31). The contribution of CNS3 in inducing mFoxp3 is distinct from these enhancers. It is thought that CNS3 interacting with cRel induces *mFoxp3* gene expression through chromatin remodeling of the *mFoxp3* gene locus in nTreg precursor cells (30).

In contrast with murine studies, the regulation of the human *FOXP3* (*hFOXP3*) gene remains poorly understood. Although the analysis of *hFOXP3* gene expression in immune mediated diseases may provide clues to clinical relevance, such information is also confounded by disease variants and distinctive features of individual patients (37). Equally, attempts to stabilize hFOXP3 expression in adoptive T-cell therapy, for both natural and induced Treg, would benefit from simple reporter readouts of *FOXP3* gene expression both *in vivo* and *in vitro*. To address these issues, we have generated hFOXP3 expression reporter transgenic mice and cell lines using BAC technology. Since BAC clones contain long DNA fragments (approximately 200-kb), gene expression from BAC DNA in transgenic mice is usually identical to that of the endogenous gene. Indeed, to control tissue specific gene expression of particular proteins (e.g., Cre recombinase), transgenic mice carrying the modified BAC DNA (e.g., insertion of the Cre recombinase cDNA into the target genes in BAC DNA) have been widely generated (38).

The resulting transgenic mouse exhibited AmCyan (hFOXP3) expression which was restricted to CD3^+^CD4^+^CD25^+^ cells in thymus and spleen, and inducible in naive CD4^+^ T-cells in a TGF-β-dependent way. In previous studies (23, 26, 28, 29, 31), we and others have characterized *mFoxp3* promoter and enhancer activities using a murine T cell line, EL4 (LAF and B02 sub-clones), which expresses mFoxp3 under similar inductive influences as those for primary mouse CD4^+^ T-cells. In order to study hFOXP3 expression *in vitro* and to screen for drugs that impact its expression, we also generated a *hFOXP3* reporter system using this cell line. Collectively, our data obtained with these reporter systems, indicate that the known murine inductive influences also control *hFOXP3* gene expression. These findings provide some basis for screening of drugs that could modulate hFOXP3 expression both *in vitro* and *in vivo*.

## Materials and Methods

### Generation of Human FOXP3 BAC Transgenic Mice

**Figure 1:**
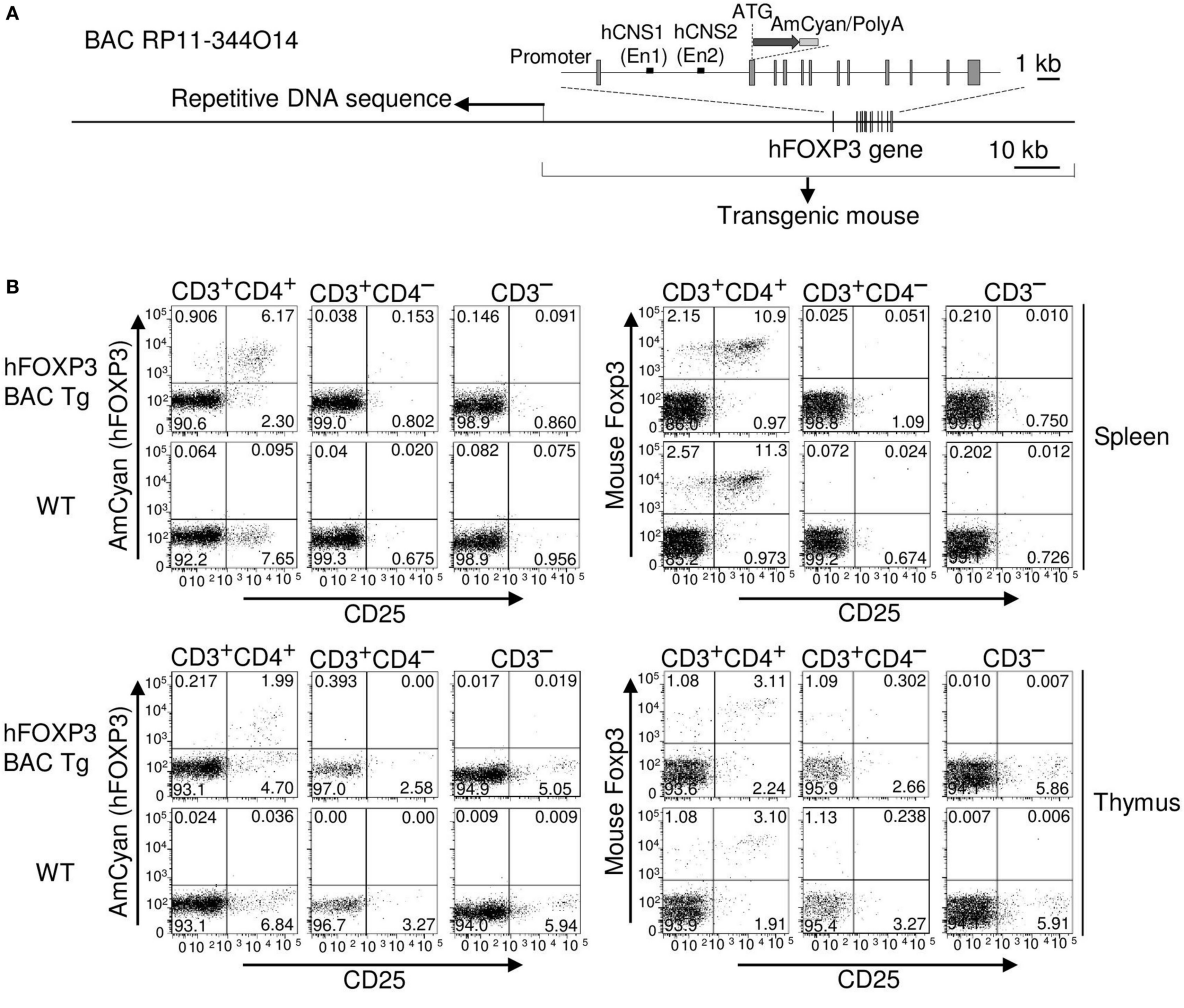

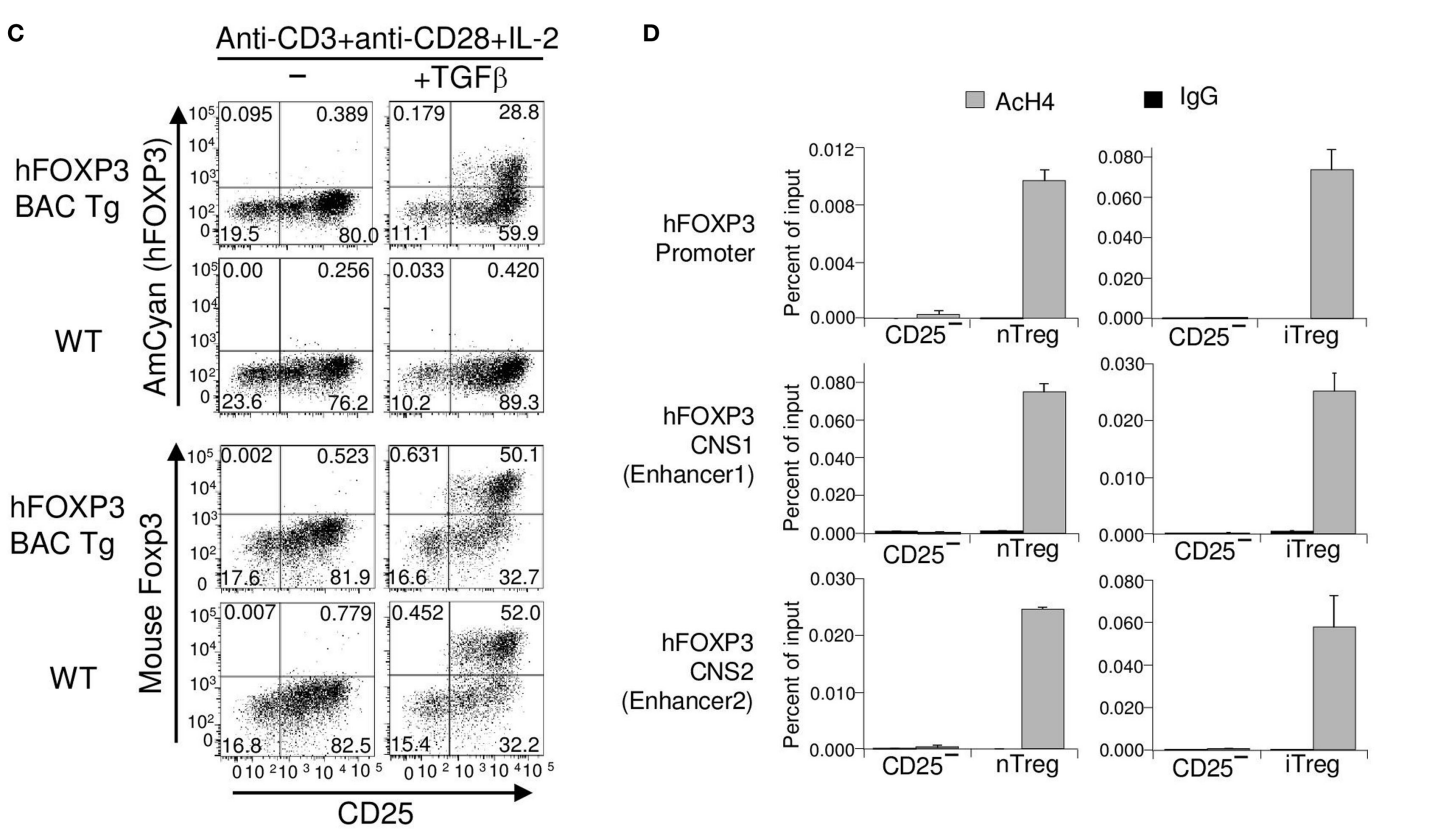
**AmCyan (hFOXP3) expression in hFOXP3 bacterial artificial chromosome (BAC) transgenic mice**. **(A)** Structure of human BAC DNA (RP11-344O14) is illustrated. Relative positions of the exons (boxes), Enhancer1 (En1) in hCNS1, Enhancer2 (En2) in hCNS2, the first ATG of the *hFOXP3* gene, and the inserted AmCyan gene are indicated. The 3′-end of the repetitive sequence in the BAC DNA is indicated by an arrow. **(B)** AmCyan (hFOXP3), mFoxp3 (internal staining), and CD25 expression in CD3^+^CD4^+^, CD3^+^CD4^−^, and CD3^−^ splenocytes and thymocytes from AmCyan/FOXP3 transgenic mice (hFOXP3 BAC Tg) and wild-type (WT) C57BL/6 mice were analyzed by flow cytometry. **(C)** AmCyan (hFOXP3) and CD25 expression in TGF-β induced Treg (iTreg) was analyzed by flow cytometry. To generate iTreg, CD4^+^CD25^−^ T cells were isolated from spleen of the hFOXP3/AmCyan transgenic mice (hFOXP3 BAC Tg) or WT C57BL/6 mice WT and stimulated with anti-CD3 + anti-CD28 + IL-2 with or without TGF-β for 72 h. **(D)** Chromatin immuno-precipitation assays were performed with CD4^+^CD25^−^, nTreg and iTreg (CD4^+^CD25^−^ T-cells stimulated with anti-CD3 + anti-CD28 + TGF-β for 48 h) enriched population using anti-acetyl histone H4 (AcH4) or control IgG and human-specific PCR primers binding to the hFOXP3 promoter, the En1 in CNS1, or the En2 in CNS2. Human specificity of the primers are shown in Figure 2B. Data are representative of three **(B,C)** or four **(D)** independent experiments (error bars indicate the SD of triplicate samples).

A human BAC clone containing *hFOXP3* gene, RP11-344O14 was purchased from Life Technologies. The 5′-half of BAC DNA was removed by homologous recombination by using RedET recombinase (39, 40) (Gene Bridges) with the homologous arm encoded in the vector and the BAC DNA located 3′-downstream region of the repetitive DNA region (Figure 1A). To replace *hFOXP3* gene expression with a fluorescent protein, AmCyan cDNA (from the first ATG) was inserted just downstream of the first ATG of hFOXP3 (the hFOXP3 first ATG was replaced by the AmCyan first ATG) by a homologous recombination procedure with modifications. BAC transgenic C57BL/6 mice were generated and bred by the Mouse Genetic Core at Cedars-Sinai Medical Center. Experimental protocols using the transgenic mice were approved by IACUC at Cedars-Sinai Medical Center (#3001&6528).

### Generation of Human and Mouse FoxP3 Expression Reporter Lines

To generate hFOXP3 expression reporter cell lines, the same modified hFOXP3 BAC was used with the addition of a neomycin resistance gene (under control of a CMV promoter). This resistance gene was inserted into the vector sequence upstream of the 5′-end of the BAC DNA. mFoxp3 expression reporter cell lines were also generated using the mouse BAC clone RP23-267C15 (Life Technologies). mFoxp3 expression from this BAC clone was replaced by the mCherry fluorescent protein by the same procedure as described for the hFOXP3 BAC modification. The resulting hFOXP3/AmCyan and mFoxp3/mCherry modified BAC are illustrated in Figures 3A and 4A, respectively. These BAC DNAs were transfected into EL4 B02 sub-clone cells by nucleofector 4D, and transfectants were selected by culture with G418 for 2 weeks. To select AmCyan (hFOXP3) and mCherry (mFoxp3) expressing cells, these transfectants were stimulated by plate-coated anti-CD3 (5 μg/ml in PBS) and TGF-β (5 ng/ml) for 48 h and AmCyan and mCherry positive cells were sorted and cloned by limiting dilution. JE6.1 transfectants using the hFOXP3/AmCyan BAC construct were also generated by the same strategy as that used for generating the EL4 reporter cells shown in Figure S1B in Supplementary Material.

### Analysis of Integrated BAC Region

**Figure 2:**
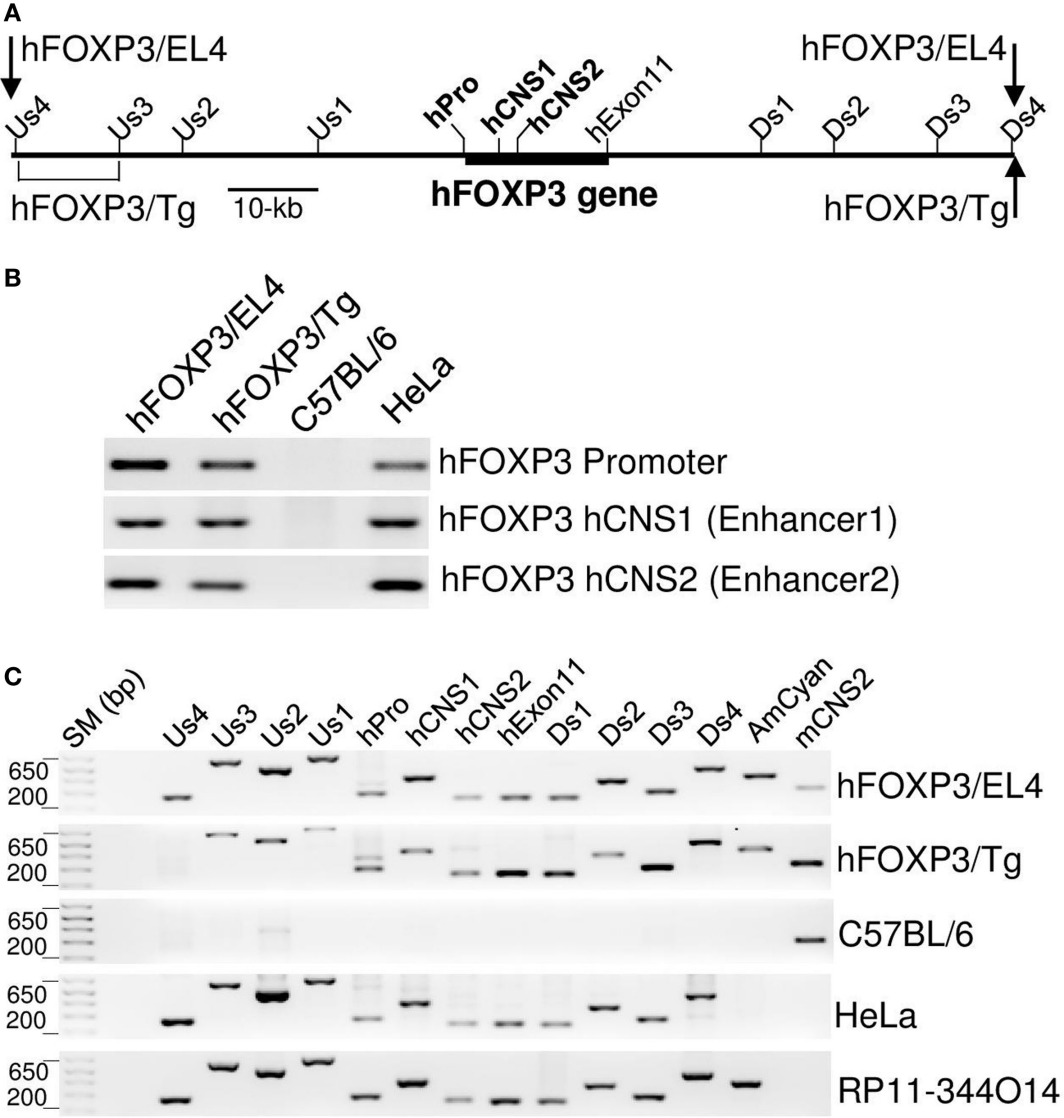
**Structure of hFOXP3 bacterial artificial chromosome (BAC) DNA**. **(A)** Relative position of the *hFOXP3* gene is indicated by thick line, positions of the hFOXP3 promoter, hCNS1, hCNS2, and the last exon (hExon11) are also indicated. PCR primer binding sites used in B and C are also indicated. **(B,C)** DNA isolated from hFOXP3/AmCyan reporter cells (hFOXP3/EL4), hFOXP3/AmCyan transgenic mouse (hFOXP3/Tg), C57BL/6 mouse, human HeLa cells, and RP11-344O14 BAC clone were analyzed by PCR. The PCR products using chromatin immuno-precipitation primers (in B) were analyzed by agarose gel and shown in **(B)**. The PCR products using primers binding to indicated positions **(A)** were analyzed by agarose gel and shown in **(C)**. AmCyan and mCNS2 primers shown in **(C)** are binding to AmCyan gene and mouse CNS2 sequences, respectively. Data are representative of four **(B,C)** independent experiments.

Hunan BAC DNA in hFOXP3/AmCyan transgenic mice and cell lines were analyzed by PCR using genomic DNA as well as DNA sequencing of the PCR products. PCR primer binding positions are shown in Figure 2A. Primer sequence used were Us4F: CCATTATTTGCCACCTCTTCGTGG, Us4R: AGTTCAGACTTGGTCCGGATGGT, Us3F: CAATCGCCCCTTCTTCACCT, Us3R: CCTCTGCGTTTTGTCACACG, Us2F: GCCTTACAAGAGCTCCTGAAGGAAG, Us2R: GGTGCTCCTGTATTGGGTGCATA, Us1F: CCGAAGCTTGCCAATTGCTT, Us1R: TGTGACTATTGCTGGGCTGG, hPROF: CGTGATTATCAGCGCACACACTCA, hPROR: CTGGGTACATCCCACTGTACCAGA, hCNS1F: GGTTGTCTGGTCATGTCCTTACTCC, hCNSR: ATCACACATAGGGCTTGGGGTGAC, hCNS2F: TGGTGTCGATGAAGCCCGGC, hCNS2R: CATGGAGATGATCTGTCTGGGGGTAG, hExon11F: GTACACTCAAACAACCTCAAAGCTGC, hExon11R: TGCATATGCGTGAGATACACA GGTG, Ds1F: ATGGTGGAGATCACCAGCAAGCA, Ds1R: CTGGTCGGATTTCGCAGCTCCTA, Ds2F: CTCCTATTCCTTCTACCCCAGAAGCT, Ds2R: CAGACAGTGTGATGATAAGAGCCTGG, Ds3F: TATTGTGGTGGGCAGCATAGTGGA, Ds3R: ACCGATGGAGAAGCCAATGGAGAA, Ds4F: GAAAGAAGTAGGCACAGCGGTGAG, Ds4R: GTGGATAAATGACG TGCCCATGAG, AmCyanF: ACATCCTGTCCACCGTGTTCATGT, AmCyanR: ATGGTCACGGGCTTCTTGGTCTTGT, mEN2F: GAAAGACAGAATCGATAGAACTTGG, and mEN2R: AATATGTTTTCCTATCGGGGTCTAC.

### Cell Culture and Reagents

EL4 sub-clone B02 cells were cultured in IMDM with l-glutamine and 25 mM HEPES (Cellgro) and 5% FBS. Primary T-cells were cultured in RPMI 1640 with l-glutamine (Cellgro) and 10% FBS. CD4^+^CD25^+^ and CD4^+^CD25^−^ T-cells from spleen were isolated using EasySep™ mouse CD4^+^CD25^+^ regulatory T-cell Isolation kit (STEMCELL). The purity of CD4^+^CD25^+^ T-cells and CD4^+^CD25^−^ T-cells was higher than 80%. mFoxp3 expression was analyzed using a Foxp3 staining kit (eBiosciences). To generate iTreg cells, CD4^+^CD25^−^ T-cells were cultured with plate-coated anti-CD3 (KT3, 5 μg/ml in PBS), anti-CD28 (1 μg/ml), recombinant human TGF-β1 (Peprotech, 5 ng/ml), and mouse IL-2 (Peprotech, 20 ng/ml). Pharmacological inhibitors, Smad3 inhibitor (SIS3, EMD Millipore), JNK Inhibitor II (SP600125, LC laboratories), CsA (Sigma-Aldrich), STAT5 inhibitor (Calbiochem, Millipore), JAK inhibitor (R545, Rigel), and Torin1 (Millipore) were used where indicated. For FACS staining, anti-CD3, anti-CD4, anti-CD25, and anti-Foxp3 (eBioscience) were used.

### Chromatin Immuno-Precipitation (ChIP)

Chromatin immuno-precipitation assays were performed using freshly isolated CD4^+^CD25^−^ T-cells, CD4^+^CD25^−^ T-cells treated with anti-CD3 + anti-CD28 + TGF-β, and CD4^+^CD25^+^Foxp3^+^ nTreg cells from hFOXP3 BAC transgenic mice, as well as EL4 transfectants as described previously (28). Briefly, these cells were fixed (for 10 min at room temperature in 1% formaldehyde, 4.5 mM HEPES pH 8.0, 9 mM NaCl, 0.09 mM EDTA, and 0.045 mM EGTA) and sonicated (Bioruptor) in lysis buffer (1% SDS, 10 mM EDTA, and 50 mM Tris–HCl pH 8.0) with proteinase inhibitor (Sigma-Aldrich P8340). Pre-cleared lysates were incubated overnight at 4°C with polyclonal anti-acetyl histone H4 (Millipore) or control rabbit IgG (Santa Cruz). DNA fragments were isolated from the immuno-precipitated chromatin and analyzed by real-time PCR with SsoAdvance SYBR supermix (Bio-Rad). PCR primers for ChIP used were hFOXP3ProF: CTGGCATTTCCCATCCACACATAGA, hFOXP3ProR: TGAGTGTGTGCGCTGATAATCACG, hFOXP3CNS1F: GGTTGTCTGGTCATGTCCTTACTCC hFOXP3 CNS1R: ATCACACATAGGGCTTGGGGTGAC, hFOXP3CNS2F: ACCCAAGAAGGGCCAGGTCTTC, and hFOXP3En2R: GCCGGGCTT CATCGACACCA.

## Results

### Human FOXP3 (AmCyan) Expression in hFOXP3 BAC Transgenic Mice

To confidently exploit knowledge of *foxp3* gene expression for therapeutic benefit, we sought to establish whether the human gene can be expressed in both transgenic mice and cell lines using an hFOXP3 BAC clone (RP11-344O14). To avoid any potential disruption of Treg development from an additional functional *hFOXP3* gene in the transgenic mice, and to provide a sensitive reporter of hFOXP3 expression, a fluorescent protein AmCyam gene was inserted into the *hFOXP3* gene in the BAC DNA by homologous recombination, resulting in the BAC construct being unable to express hFOXP3 protein (hFOXP3 expression is replaced by AmCyan) (Figure 1A). During the BAC DNA modification process, we found a long repetitive DNA region located in the 5′-half of the clone (Figure 1A). Since this repetitive sequence interfered with the modification process, we removed the 5′-half of the BAC DNA sequence. The resulting BAC, therefore, contains 52 and 59 kb DNA sequences at the 5′- and 3′-flanking regions of the hFOXP3 transcription start site, respectively. To replace hFOXP3 expression with AmCyan, cDNA encoding this fluorescent protein was inserted just downstream of the first ATG of the *hFOXP3* gene (the first ATG of the *hFOXP3* gene was replaced by that of the AmCyan cDNA, resulting no hFOXP3 protein expression) (Figure 1A). C57BL/6 transgenic mice were generated using the modified BAC DNA, and AmCyan (hFOXP3) expression was analyzed by FACS.

### AmCyan (hFOXP3) Expression Was Restricted to CD3^+^CD4^+^CD25^+^ Cells and TGF-β Induced Treg

Expression of both AmCyan and endogenous mFoxp3 was detected only within CD3^+^CD4^+^ cells (but not in CD3^−^ cells nor other CD3^+^ cells such as CD3^+^CD8^+^ cells) and was restricted to CD4^+^CD25^+^ cells from the thymus and spleen of the BAC transgenic mice. No AmCyan expression could be detected in wild-type (WT) mice (Figure 1B). This tells us that the developmental pathway for mouse nTreg, can provide all the cues for BAC hFOXP3 expression. However, although cell type expression patterns of AmCyan and mFoxp3 were similar, the percentage of mFoxp3^+^ cells was higher than that expressing AmCyan^+^. Since the ratio of AmCyan^+^ compared to mFoxp3^+^ cells was similar in spleen and thymus (in Spleen, AmCyan^+^ 6.17% vs. mFoxp3^+^ 10.9% and in Thymus, AmCyan^+^ 1.99% vs. mFoxp3^+^ 3.11%), hFOXP3 induction in tTreg precursor cells appeared to be somewhat less efficient than for the endogenous gene.

To examine the performance of the BAC construct in murine iTreg, we examined the effect of TGF-β in regulating hFOXP3 expression in *in vitro*. CD4^+^CD25^−^ T-cells were isolated from spleens of the hFOXP3 BAC transgenic and WT mice, and cultured with anti-CD3 + anti-CD28 + IL-2 with or without TGF-β (Figure 1C). Expression of AmCyan (hFOXP3) and mFoxp3 (internal staining) was observed only in cells cultured with TGF-β, indicating that *hFOXP3* gene expression is also TGF-β dependent. Yet again, however, the percentages of TGF-β-induced hFOXP3^+^ iTreg from the BAC transgenic mice (28.8%) were lower than mFoxp3^+^ iTreg generated from WT (52.0%) and the BAC transgenic mice (50.1%). Taken together, these data suggest that fundamentally, *hFOXP3* and *mFoxp3* genes behave similarly in the murine inductive conditions, but that hFOXP3 (AmCyan) expression is somewhat constrained. This could be due to features of the transgenic mice generated with the engineered BAC DNA construct or to transcriptional activity of gene expression for hFOXP3 being weaker than that for mFoxp3. It is also possible that the Foxp3 staining process, in some way, inhibits AmCyan intensity. We, therefore, further investigated whether the regulatory regions identified for *mFoxp3* expression also function for the *hFOXP3* gene. As shown in previous studies (18–30), *mFoxp3* transcription is regulated by three regulatory regions located in the promoter, CNS1 and CNS2. Many histone modifications can be used to assess the accessibility of chromatin in these regions by ChIP assay using anti-acetyl-histone and anti-methyl-histone (e.g., H3K27ac, H3K27me3, H3K9me3, and H4ac). We performed ChIP assays using anti-acetyl histone H4 and human-specific PCR primer sets to compare with the previous mFoxp3 ChIP data obtained using the same antibody (28, 31). The relative positions of the promoter, human CNS1 (hCNS1), and hCNS2 are shown in Figure 1A and Figure 2A, and human specificity of the primers for ChIP assay was shown in Figure 2B. Histone H4 molecules in the hFOXP3 promoter (hPro), hCNS1, and hCNS2 were highly acetylated in both the nTreg and iTreg (CD4^+^CD25^−^ T cells stimulated with anti-CD3 + anti-CD28 + TGF-β for 48 h) enriched populations, but not in the control freshly isolated CD4^+^CD25^−^ T-cells (Figure 1D). This pattern was identical to that in *mFoxp3* gene as previously shown (28, 31). Taken together, these data suggest that mFoxp3 and hFOXP3 induction can be regulated by similar mechanisms through the promoters, hCNS1 and hCNS2.

### Generation of Human and Mouse *foxp3* Gene Expression Reporter Cells

**Figure 3:**
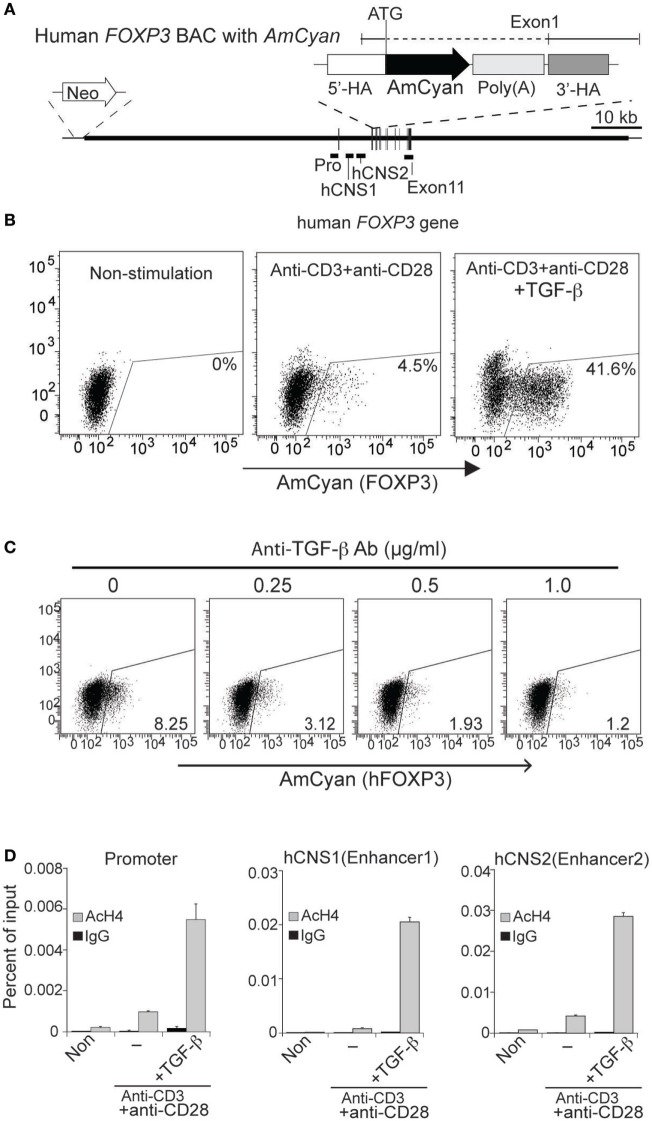
**Generation and characterization of hFOXP3/AmCyan expression reporter cells**. **(A)** Structures of hFOXP3/AmCyan bacterial artificial chromosome (BAC) DNA are illustrated. Relative positions of hFOXP3 promoter, hCNS1, and hCNS2 are shown. AmCyan gene was inserted in the *hFOXP3* gene by homologous recombination using the indicated donor fragment with 5′and 3′-homologouse arms and poly(A) additional sequence. Neomycin-resistant gene is inserted at the 5′-end of the BAC DNA. **(B)** hFOXP3/AmCyan reporter cells were stimulated (for 48 h) with no stimuli (Non-stimulated), anti-CD3 + anti-CD28 without or with TGF-β, and AmCyan (hFOXP3) expression was analyzed by flow cytometry. **(C)** hFOXP3/AmCyan reporter cells were stimulated with anti-CD3 + anti-CD28 and cultured with anti-TGF-β (with indicated concentration). AmCyan expression was analyzed by flow cytometry. **(D)** Chromatin immuno-precipitation assay was performed using anti-acetyl histone H4 (AcH4) or control IgG and non-stimulated (Non) cells, or stimulated cells by anti-CD3 + anti-CD28 without (−) or with (+) TGF-β. Human specificity of the used primers binding to the promoter, hCNS1 and hCNS2 are shown in Figure 2B. Data are representative of four **(B)**, three **(C)**, or four **(D)** independent experiments (error bars indicate the SD of triplicate samples). Three **(B)** and two **(C)** independent clones were also analyzed.

**Figure 4:**
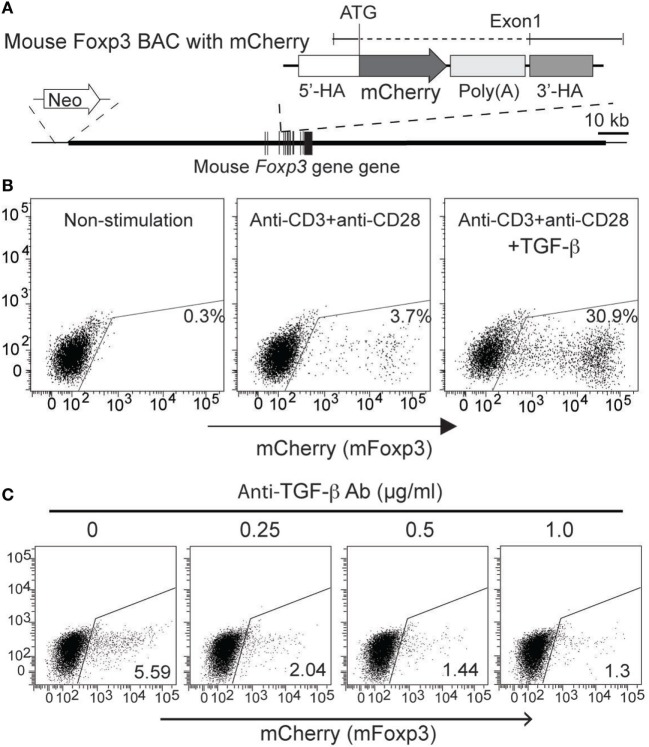
**Generation and characterization of mFoxp3/mCherry expression reporter cells**. **(A)** Structures of mFoxp3/mCherry BAC DNA are illustrated. mCherry gene was inserted in the *mFoxp3* gene by homologous recombination with the similar strategy shown in Figure 3A. **(B)** mFoxp3/mCherry reporter cells were stimulated (for 48 h) with no stimuli (non-stimulated), anti-CD3 + anti-CD28 without or with TGF-β, and mCherry (mFoxp3) expression was analyzed by flow cytometry. **(C)** mFoxp3/mCherry reporter cells were stimulated with anti-CD3 + anti-CD28 and cultured with anti-TGF-β (with indicated concentration). mCherry expression was analyzed by flow cytometry. Data are representative of four **(B)** or three **(C)** independent experiments. Four **(B)** independent clones were also analyzed.

The data obtained using hFOXP3/AmCyan transgenic mice suggested that both human and mouse *foxp3* gene expression can utilize identical regulatory regions in the corresponding genes. To investigate mouse and human *foxp3* gene expression further, and to establish a screening system for drugs influencing *hFOXP3* gene expression, we generated a hFOXP3 expression reporter cell line using hFOXP3/AmCyan BAC DNA and EL4 sub-clone B02. As we have previously shown (28, 31), both EL4 sub-clones LAF and B02 expressed mFoxp3 after stimulation with anti-CD3 ^+^ anti-CD28 + TGF-β, indicating that mFoxp3 expression in these sub-clones is induced by TCR activation and TGF-β signaling as in TGF-β induced Treg, unlike the original EL4 cell line. Furthermore, using these sub-clones, we and others have identified diverse transcription factors involved in *mFoxp3* gene expression (23, 26, 28, 29, 31). Therefore, we generated hFOXP3/AmCyan BAC (Figure 3) and mFoxp3/mCherry BAC (Figure 4) transfectants using EL4 B02 cells, as hFOXP3 and mFoxp3 expression reporter systems. To select BAC transfectants, the neomycin-resistant gene was inserted at the 5′-end of the BAC DNA (Figures 3A and 4A). Although BAC transfectants were selected by G418 as neomycin resistant, many transfectants might, in principle, have lost the *foxp3* gene and/or essential transcriptional regulatory regions for FoxP3 expression during the BAC DNA integration process. To select for FoxP3 expressing transfectants, these G418 resistant cells were stimulated with anti-CD3 + TGF-β for 48 h and AmCyan and mCherry positive cells were sorted and cloned by limiting dilution. During this cloning step (culturing without stimuli for about 2 weeks), stimulated cells reverted to the “resting” state and did not express these fluorescent proteins without further stimulation.

Since consistent AmCyan (*hFOXP3*) gene expression in the hFOXP3/AmCyan transgenic mice could be detected in appropriate cell types, it appears that mouse and human *foxp3* genes exploit common regulatory mechanisms. However, reporter systems using human cell lines would, if available, enable identification of any human-specific regulatory mechanisms. To this end, we have sought human T-cell lines that regulate inducible hFOXP3 expression akin to iTreg. None have been described. However, since we can stimulate the human T cell line JE6.1 with anti-CD3 and TGF-β (41, 42), we generated transfectants using the same hFOXP3/AmCyan BAC construct (Figure 3A) with the strategy that had proven successful for EL4 B02 cells (Figure S1 in Supplementary Material). However, the expression pattern of endogenous hFOXP3 mRNA from JE6.1 was unlike iTreg. hFOXP3 mRNA was detected in non-stimulated cells, yet not significantly upregulated by stimulation (Figure S1A in Supplementary Material). Importantly, AmCyan (hFOXP3) expression from the BAC DNA in JE6.1 transfectants was similar to that of the endogenous *hFOXP3* (Figure S1C in Supplementary Material), but dissimilar to iTreg and reporter cells generated using the EL4 B02 line. In short, the expression of the *foxp3* gene in Treg seems not only to be determined by regulatory regions in this gene but also by other, as yet, undefined regulatory mechanisms. The result suggests although EL4 B02 line is a mouse cell line, it remains the best option for a reporter system to date, albeit not covering as putative set of human-specific regulatory mechanisms.

### TGF-β-Mediated AmCyan (hFOXP3) and mCherry (mFoxp3) Induction in the Reporter Cells

To examine whether expression of AmCyan (hFOXP3) and mCherry (mFoxp3) in BAC transfectants was similar to mouse primary T-cells, cloned transfectants were re-stimulated by anti-CD3 + anti-CD28 with or without TGF-β and analyzed by FACS. The expression patterns of both AmCyan (hFOXP3) (Figure 3B) and mCherry (mFoxp3) (Figure 4B) in these transfectants were similar, and induction of both AmCyan and mCherry was TGF-β dependent, just as in hFOXP3 BAC transgenic mice and WT mice (Figure 1C). However, 3 to 8% of AmCyan^+^ and mCherry^+^ cells were always detected in cultures stimulated with anti-CD3^+^ anti-CD28, but without additional TGF-β (4.5% in Figure 3B and 3.7% in Figure 4B). This residual AmCyan and mCherry induction was, however, inhibitable by addition of anti-TGF-β (Figures 3C and 4C), implicating other sources of TGF-β as responsible (e.g., TGF-β expressed by EL4 cells themselves and/or bovine TGF-β in FBS).

We, next, examined whether the three regulatory regions identified in the hFOXP3/AmCyan transgenic mice (promoter, hCNS1 and hCNS2) (Figure 1D) and WT mice (28, 31) are also involved in induction of hFOXP3 expression in the EL4 reporter cells. We assessed the “open chromatin status” of these regulatory regions by ChIP assay (Figure 3D) using anti-acetyl histone H4 and the human-specific PCR primers (Figures 2A,B). As with the hFOXP3/AmCyan transgenic mice (Figure 1D), histone H4 molecules in the hPro, hCNS1, and hCNS2 were highly acetylated only in anti-CD3 + anti-CD28 + TGF-β stimulated cells, suggesting that hFOXP3 expression in the EL4 reporter cells could also be induced through these three regulatory regions with TCR and TGF-β signaling. These data suggest that the EL4 reporter system should be useful for further studies of hFOXP3 expression and identification of potential therapeutics in modulating hFOXP3 expression.

### The Regulatory Regions Controlling Cell Type-Specific hFOXP3 Expression in Treg Are Located within the 110-kb BAC DNA Sequence

The regulation of hFOXP3/AmCyan expression in the reporter transgenic mice and transfectants appeared similar to that previously seen in mouse primary T-cells. This indicates that the human BAC DNA in both transgenic mice and transfectants contains all relevant regulatory regions required for Treg specific *hFOXP3* gene expression in murine cells. We analyzed the integrated area of the hFOXP3 BAC DNA in the transgenic mice and transfectants by PCR using human-specific primer sets (relative positions of these primer binding sites are shown in Figure 2A). As shown in Figure 2C, strong amplification bands were detected with the human-specific PCR primer sets using DNA from HeLa cells and the BAC clone (RP11-344O14) but not using DNA from WT C57BL/6 mice (only weak non-specific bands were detected with some of these primers). In the transgenic mice, a strong PCR band was detected with the Us3 primer set (binding to −40 kb from the transcription start site of the hFOXP3 gene), but not with the Us4 primer (binding to −51 kb), implicating the 5′-end of the hFOXP3 BAC DNA located between −51 and −40 kb (Figures 2A,C). Since the neomycin resistant gene is located at the 5′-end of the hFOXP3 BAC DNA (Figure 3A), the hFOXP3/AmCyan EL4 transfectant contains the 5′-end of the BAC DNA, and indeed, a strong 5′-band was also detected with the most 5′-PCR primer set (Us4) (Figures 2A,C). In both the transgenic mice and the transfectants, strong PCR bands were detected with the most 3′-PCR primer set, Ds4, indicating that both 3′-ends of the integrated DNA are located near the 3′-end of the BAC DNA (Figure 2C). Taken together with the results of Treg-specific hFOXP3/AmCyan gene expression in the transgenic mice, our data suggests that the regulatory regions required to maintain Treg-specific *hFOXP3* gene expression are located within the 110-kb (−51 to +59 kb) DNA sequence area shown in Figure 2A.

### Transcription Factors Involved in *hFOXP3* Gene Expression

**Figure 5:**
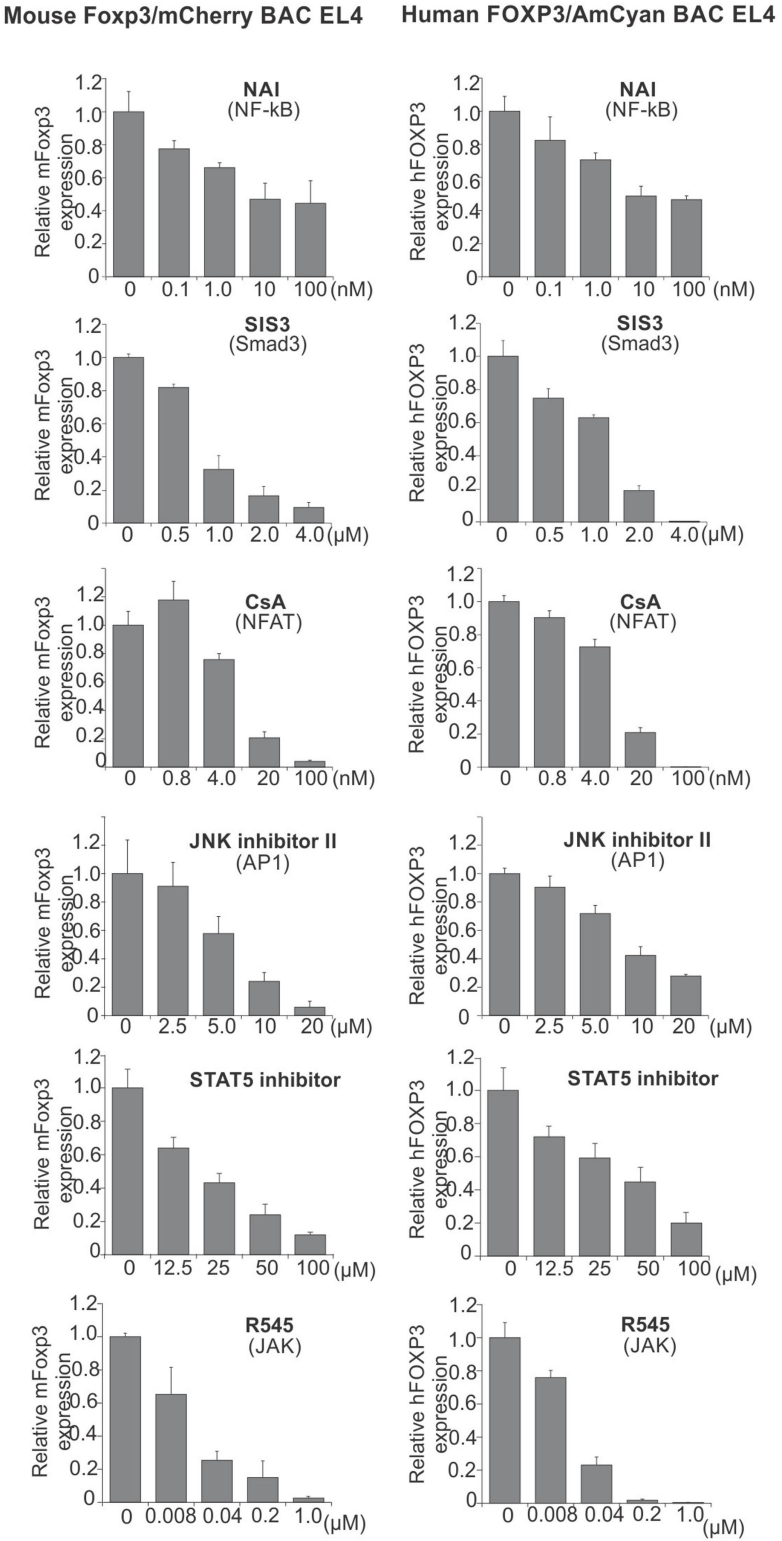
**Inhibition of AmCyan (hFOXP3) and mCherry (mFoxp3) expression**. mFoxp3/mCherry or hFOXP3/AmCyan reporter cells were cultured with different concentrations of the indicated transcription factor inhibitors, NAI (NF-κB activation inhibitor), SIS3 (Smad3 inhibitor), CsA (NFAT inhibitor), JNK inhibitor II (AP1 inhibitor), STAT5 inhibitor, and R545 (new JAK inhibitor) for 48 h. mCherry (mFoxp3) or AmCyan (hFOXP3) expression was analyzed by flow cytometry and relative expression levels are shown. Data are representative of three independent experiments (error bars indicate the SD of triplicate samples).

We have previously shown that NF-κB cRel (in promoter) (26), Smad3 (in CNS1) (28), NFAT (in CNS1) (28), AP1 (in CNS2) (31), and STAT5 (in CNS2) (31) cooperate to regulate *mFoxp3* gene expression, and that pharmacological inhibitors of the activation pathways for these transcription factors blocked *mFoxp3* gene expression. In order to provide a proof of principle test that our hFOXP3 construct might serve as a tool to test drugs for induction or repression of Foxp3 transcription, we examined the contribution of these transcription factors in regulating *hFOXP3* gene expression. To this end, we used these same inhibitors in the context of the hFOXP3/AmCyan expression system (Figure 5). mFoxp3 expression was also analyzed using the mFoxp3/mCherry expression system as a control. Similar to mCherry (mFoxp3) expression, AmCyan (hFOXP3) expression was blocked by the inhibitors of these transcription factors. Taken together with ChIP data shown in Figures 1D and 3D, these findings suggest that *hFOXP3* gene expression is also regulated by the same transcription factors through the promoter, hCNS1 and hCNS2.

Since downmodulation of Foxp3 expression by inhibitors for NFAT, Smad3, AP1, and STAT5 has previously been described in primary iTreg (28, 29, 31), the reporter cells seem useful for identifying some drugs that can control *hFOXP3* gene expression in these cells and primary T cells. To further support the potential of this system for identifying drugs able to influence *hFOXP3* gene expression, we assessed a newly synthesized JAK inhibitor (R545) (43). Since mouse and human *foxp3* gene expression is regulated by STAT5, the JAK inhibitor would be expected to downmodulate gene expression through blocking phosphorylation of STAT5. Indeed, expression of both *foxp3* genes is strongly inhibited by R545, suggesting that these reporter systems hold promise for screening drugs modulating hFOXP3 expression.

### hFOXP3 Expression Is Enhanced in the Reporter Cells by Treatment with the mTOR Inhibitor, Torin1

**Figure 6:**
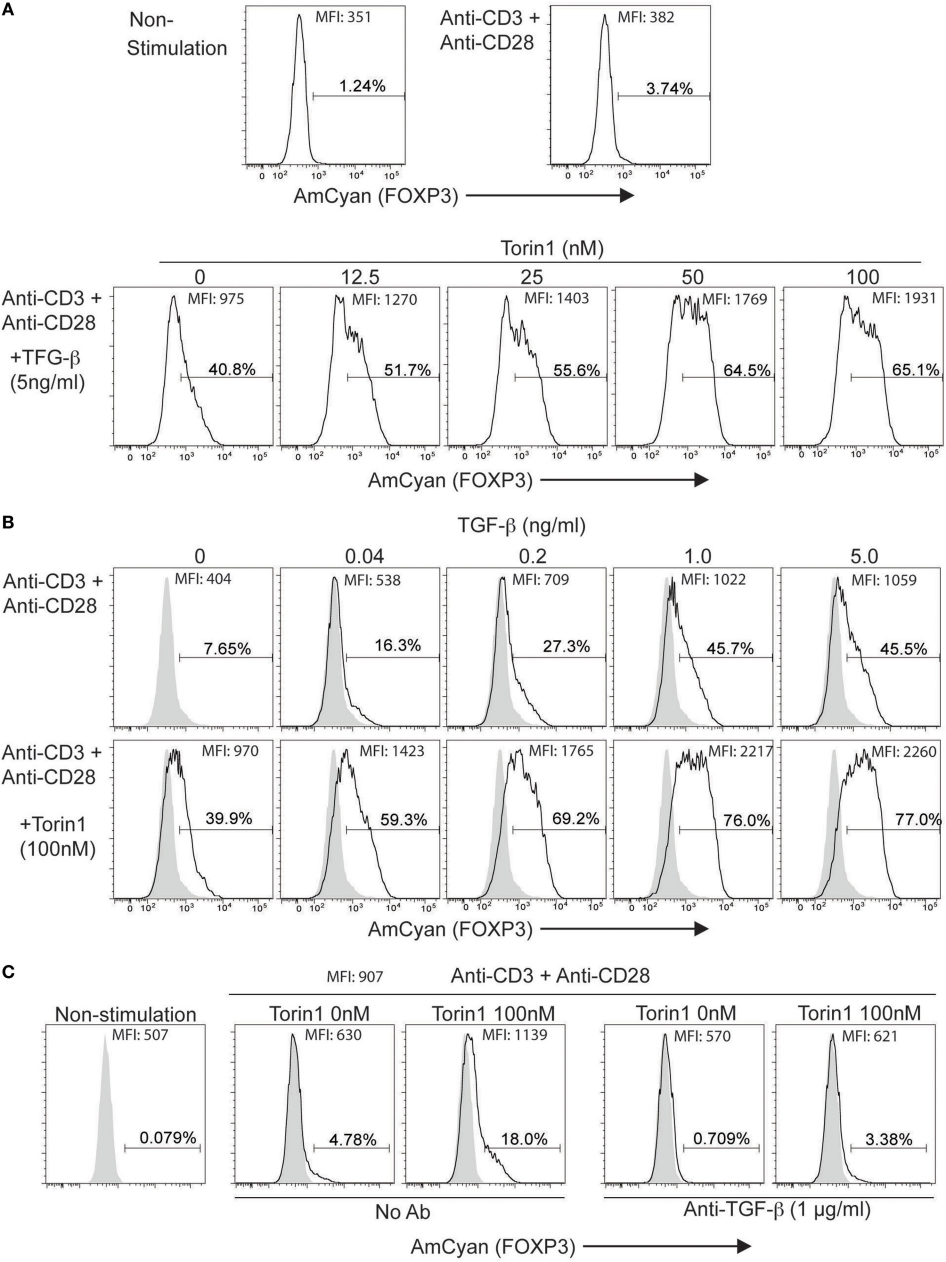
**Enhancement of AmCyan (hFOXP3) expression by Torin1**. AmCyan^+^ cells were analyzed by flow cytometry, and percentages of positive cells and mean fluorescence intensity are indicated. **(A)** hFOXP3/AmCyan reporter cells were cultured with no stimuli (non-stimulation) or with anti-CD3 + anti-CD28 (Anti-CD3 + Anti-CD28). AmCyam/hFOXP3 reporter cells were also stimulated by anti-CD3 + anti-CD28 + TGF-β (5 ng/ml) with indicated concentrations (0–100 nM) of Torin1 for 48 h. **(B)** hFOXP3/AmCyan reporter cells were stimulated by anti-CD3 + anti-CD28 without or with (100nM) Torin1 with indicated concentrations of TGF-β. **(C)** hFOXP3/AmCyan reporter cells were cultured with no stimuli (non-stimulation) or with anti-CD3 + anti-CD28 for 48 h. Torin1 and/or anti-TGF-β were added into the culture with indicated concentration. Data are representative of four **(A)**, four **(B)**, or three **(C)** independent experiments.

Next, we sought evidence for drug-induced enhancement of hFOXP3 expression using this reporter system. Since Foxp3 expression is inhibited by the mTOR signaling pathway antagonizing the function of Smad3, Foxo1, and Foxo3 (44, 45), we examined the effectiveness of an inhibitor (Torin1) (44, 46, 47) of mTORC1 and mTORC2 using the stimulated (anti-CD3 + anti-CD28 + 5 ng/ml TGF-β) hFOXP3/AmCyan reporter cells with different concentrations of Torin1 (0–100 nM) (Figure 6A). AmCyan expression was induced in 40% of the reporter cells without Torin1, and increased with Torin1 in a dose-dependent manner. Since mTORC1 inhibits TGF-β-mediated Smad3 activity, the effectiveness of Torin1 (100 nM) was assessed using different dose of TGF-β (0–5 ng/ml). As shown in Figure 6B, using 100 nM of Torin1, AmCyan^+^ cells could be detected following exposure to low doses of TGF-β, and numbers increased substantially with higher doses of TGF-β. Although 39.9% of Torin1 treated cells and 7.65% of non-treated cells were detected as AmCyan^+^ cells without addition of TGF-β, induction of these cells was inhibited, as before, by anti-TGF-β (Figure 6C), indicating enhancement of hFOXP3 expression is completely TGF-β dependent.

## Discussion

Research on *Foxp3* gene expression has contributed significantly to our understanding of the biology of Treg. To date, most such studies have been conducted in mice facilitated through the availability of genetically modified mice and disease models. However, recent studies have suggested that the mouse and human *foxp3* genes may be regulated differently possibly minimizing the applicability of the rapidly accumulating murine data. For example, unlike mouse T-cells, hFOXP3 expression in human T-cells can be induced without the apparent need for TGF-β. It is not clear what these disparities are due to, and has left a question mark around the mouse data. In an attempt to clarify the situation, we transferred the *hFOXP3* gene with regulatory elements to mouse by creating transgenic mice using human FOXP3 BAC reporter constructs. We observed that the cellular distribution and inducibility of the reporter AmCyan (hFOXP3) expression was similar to mFoxp3 expression in these BAC transgenic mice. This suggest that, at least in part, mouse and human *foxp3* gene expression is regulated by the common mechanisms and the hFOXP3 expression reporter system can be utilized for studying both fundamental and clinically applicable mechanisms. The major impact of this reporter system is that (i) we can use the transgenic mice to study *hFOXP3* gene expression under defined experimental conditions *in vivo* and (ii) we can assess the effects of potential disease modifying drugs for their impact on hFOXP3 expression in the reporter transgenic mice, and in mice derived by crossing the reporter transgenic mice to disease-prone animals. As shown in Figures 5 and 6, EL4/hFOXP3/AmCyan system is useful to screen potential drugs to modulate *hFOXP3* gene expression. The identified potential drugs can be analyzed using the hFOXP3/AmCyan transgenic mice (*in vitro* and *in vivo*), and then, in due course, their efficacy confirmed on human Treg. With the transgenic mice data obtained in the more homogenous cell population, we may be able to avoid artifacts seen in the heterogeneous populations of human Treg.

The disadvantage of this transgenic mouse reporter system is that it is not suitable as a first-line simple screen for potential drugs that might control hFOXP3 expression and, by implication, Treg activity. To resolve this problem, we generated a hFOXP3 expression reporter cell line. The advantage of this is that we can provide large numbers of cells for drug screening, and can exploit transfection-based methods to study *hFOXP3* gene expression. Its disadvantage is that since EL4 B02 is of murine origin, we cannot identify drugs targeting human-specific mechanisms, if such exist. However, all our data obtained using hFOXP3/AmCyan reporter transgenic mice and cell lines indicate that a substantive degree of *hFOXP3* gene expression can be regulated by mechanisms similar to those influencing *mFoxp3* gene expression, as might be predicted from the finding that the *foxp3* gene structures are highly conserved in all mammals (48).

*mFoxp3* gene expression is regulated by a promoter and two enhancers located in CNS1 and CNS2, and histone H4 molecules in these regions in Foxp3 expressing cells are highly acetylated, indicating open chromatin status in these regions. Importantly, ChIP data suggest that these regions in the *hFOXP3* gene are also opened in nTreg and iTreg in the reporter systems, suggesting that these regions also function as promoter and enhancers in humans. NF-κB cRel bind to the promoter, Smad3, NFAT, and AP1 bind to CNS1, and STAT5 bind to CNS2 in the *mFoxp3* gene. Inhibitors of these transcription factors strongly downmodulated *hFOXP3* gene expression. Taken together, these findings suggest that *hFOXP3* gene expression is also regulated by these same transcription factors through these same regulatory regions. A deeper future analysis of chromatin regulation with the hFOXP3 transgene by analysis of histone marks, such as H3K27ac, H3K27me3 and H3K4, and K9 modification, will allow detailed comparison of the transgene regulation with primary human T-cells.

As shown in Figure 1C, only half of CD4^+^ primary T-cells induced mFoxp3 after stimulation with TGF-β. First, we thought that the partial induction of *mFoxp3* gene expression was caused by heterogeneity of CD4^+^ T-cells (e.g., only half of CD4^+^ T-cells can be developed to Treg precursor cells). However, clonal experiments using the FoxP3 expression reporter cells suggested the presence of inhibitory mechanisms operating in the negative cells. AmCyan (hFOXP3) and mCherry (mFoxp3) expression reporter cell lines were cloned by limiting dilution, to ensure that we only studied pure clones. Despite this, only 30–42% of the cloned cells induced AmCyan and mCherry (Figures 3B and 4B). This suggests that hFOXP3 and mFoxp3 induction are somehow constrained in the majority of the cells, even though all cells have same genetic background and received the same TCR/TGF-β signaling. Since an mTOR inhibitor enhanced the numbers of cells induced, and their expression levels, one of the constraining mechanisms may be through the mTOR signaling pathway. Alternatively, *foxp3* gene expression in the negative cells might be blocked by some epigenetic modification such as DNA methylation. In the hFOXP3/AmCyan BAC transgenic mice, endogenous mFoxp3^+^ cells were more numerous than AmCyan^+^ cells. This difference may have been due to the additional technical processing required for mFoxp3 internal staining. Future crosses of the hFOXP3 BAC transgenics with mFoxp3-IRES-GFP mice may help to clarify this issue. Alternatively, it is possible that mFoxp3^+^AmCyam^−^ cells do exist and are a consequence of other epigenetic influences selectively affecting the hFOXP3 BAC gene. Any such human FOXP3 specific regulatory mechanisms could, in due course be identified by detailed analysis of DNA methylation and histone modifications.

As shown here, the fundamental regulatory mechanisms of mouse and human *foxp3* gene expression seem to be similar. However, induction of hFOXP3 (AmCyan) was always less than that of mFoxp3 in the AmCyan/hFOXP3 expression reporter transgenic mice. This feature may be due to the integrated position of the BAC DNA, and/or unidentified human or mouse specific regulatory regions (e.g., enhancer and silencer) which also control the *foxp3* gene expression. Alternatively, different expression levels may be controlled by human or mouse specific epigenetic regulatory mechanisms. Overall, however, the hFOXP3/AmCyan expression reporter system appears useful in identifying physiological factors and drugs influential to hFOXP3 expression, and may have utility in determining why Treg may, sometimes, fail to prevent immunopathology in diverse disease models.

## Author Contributions

MTsuda and YT generated the BAC constructs and the transfectants and analyzed the reporter cells and the transgenic mice. CO performed inhibitory experiments using the reporter cells. YN and MK generated the transgenic mice. AN, DH, and YT performed experiments using Torin1. EM provided R545 and its information, MTsuda, YT, DH, HW, and MTone were involved in writing of the paper.

## Conflict of Interest Statement

The authors declare that the research was conducted in the absence of any commercial or financial relationships that could be construed as a potential conflict of interest.
