# Supplementary material for: A Bacterial Artificial Chromosome Reporter System for Expression of the Human FOXP3 Gene in Mouse Regulatory T-Cells
Source: Front Immunol. 2017 Mar 13;8:279. doi: 10.3389/fimmu.2017.00279 (PMC5346934; doi:10.3389/fimmu.2017.00279)
Supplement: Supplementary file 1 [file Presentation_1.PDF]

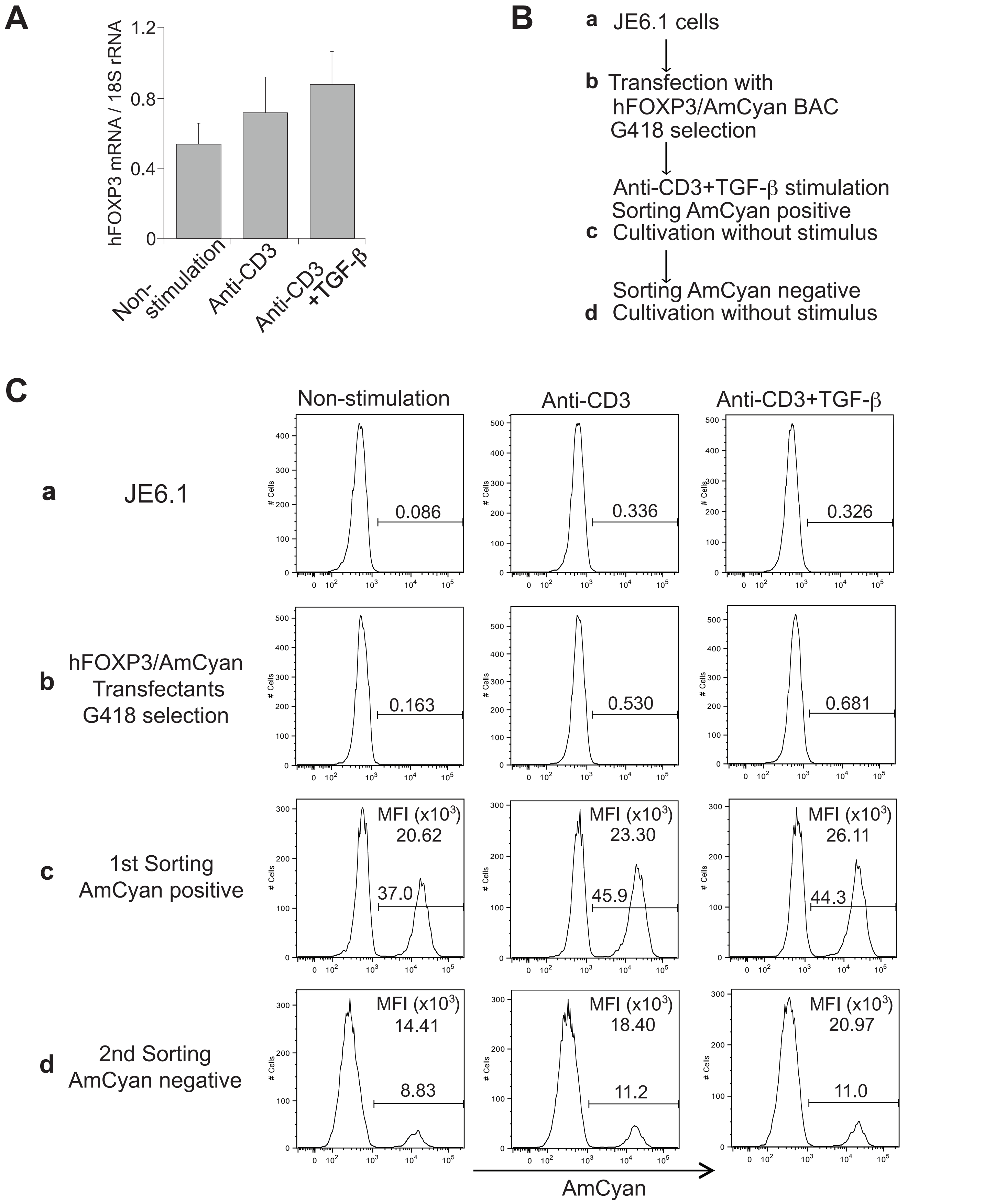

FIGURE S1. hFOXP3/AmCyan expression in human T cell line JE6.1. (**A**) JE6.1 cells were stimulated with no stimulus (Non-stimulation), anti-CD3 and anti-CD3+TGF-β for 48h. Endogenous hFOXP3 mRNA expression was analyzed by RT-PCR (normalized by 18S rRNA). (**B**) The experimental strategy to generate hFOXP3/AmCyan BAC transfectants is shown. Cells indicated by a. b. c. and d. are analyzed by FACS in C. (**C**) Indicated cells (a, b, c, d) in B were stimulated with no stimulus (Non-stimulation), anti-CD3 and anti-CD3+TGF-β for 48h, and AmCyan (hFOXP3) expression was analyzed by FACS. Percentages of positive cells and mean fluorescence intensity (MFI) are indicated.
